# Filling Teeth over Exposed Nerves

**Published:** 1851-07

**Authors:** W. H. Elliot

**Affiliations:** Member of the Medico-Chirurgical Society of Montreal.


### ARTICLE V.

*Filling Teeth over Exposed Nerves.* By W. H. ELLIOT, D. D. S., *Member of the Medico-Chirurgical Society of Montreal.*

IT is not the writer's intention in the present instance, to enter into the details of the operation of filling teeth on the principles that govern it, except so far as is necessary to explain his practice in cases in which it becomes necessary to fill over the exposed and living nerve. He was not aware, until he read the interesting discussions which took place at the last meeting of the American Society of Dental Surgeons, that his practice in this particular was so unlike that of other dentists, or at least of those whose methods were made known on that occasion. This difference consists principally in his laying the gold in all cases directly upon the living nerve, and in perfect contact with it, over the whole of its exposed surface.

The writer believing, that when any space is left between the bottom of the filling and the surface of the nerve pulp, the least increase of circulation forces the delicate living membrane of the tooth through the opening, in the partition which separates the natural from the artificial cavity, and that the sharp and irregular edge of the bone around this opening lacerates the membrane, and increases tenfold the slight but unavoidable irritation which follows the exposure of the nerve; this, or any other accident which might call a little more than the usual quantity of blood into the vessels of the nerve, would have the same effect. It is upon this belief that his present practice if founded, and it gives him much pleasure to state, that it has been far more successful in his hands than any other, and he has no doubt that it would be so in other hands if fairly tested.

The statement made by Dr. Rich before the society, that "with a good light, a steady and delicate hand, the membrane can be exposed five times out of six without hemorrhage" is perfectly correct. When this part of the operation has been successfully performed, and the gold properly introduced with a good non-conductor under it, there is nothing to fear from the operation itself.

The writer's method is as follows: He first prepares the orifice and walls of the cavity for the gold, and then removes the softened bone from the bottom of it, as far as practicable, without interfering with the nerve, with the ordinary instrument; but the excavator with which this part of the operation is completed, and the nerve laid bare, should be made, firstly, as light as possible, the vibrations of a heavy instrument are liable to turn down minute spicular into the nervous pulp from the edge of the bony partition. Steel handles will not do. Secondly, the point of the instrument should be curved to an angle of about forty-five degrees from a line with the handle, and when in use, it should be *drawn lightly* over the bone, not pushed, and always in a direction from the neck of the tooth towards the grinding surface; if the cavity be on the top of the tooth, the instrument must be then drawn from the centre of the cavity towards the walls, because it always cuts the bone more smoothly, and with less pain in this direction than in any other. Thirdly, the point of the excavator for large cavities should be at least one-eighth of an inch in width, so that in passing over the exposed nerve it would be too wide to drop into it, the edge should be slightly curved, highly tempered, and as sharp and smooth as it is possible to make it on the finest stone. Under such an edge, the nerve may be distinctly seen to yield without being injured by it, while the surrounding bone is carried away.

As a non-conductor, when the cavity is comparatively deep, the writer prefers asbestos, *enveloped in a few thicknesses of*

*gold foil,* formed into a yielding cap, sufficiently large to retain its position; this should be gently but firmly pressed down with a plugger large enough, if possible, to cover the whole of the cap, and warmed to the temperature of the tooth.

Whatever the non-conductor may be, there should always be one or two thicknesses of foil under it so as to secure a smooth surface of gold in contact with the nerve; from such a surface the nerve will bear considerable pressure without evil consequences, while the slightest spicular of bone turned into it from the edge of the partition is sure to destroy it.

Within the last year the writer numbers but two cases in which irritation advanced so far as to become troublesome to the patient; in both instances, perfect and permanent relief was obtained by the use of leeches and a mild cathartic.

There can be no doubt that the time is near at hand when the treatment of exposed dental nerves will be, in some degree, revolutionized. The desire for improvement, which characterises the leading spirits of the dental profession at the present day, will not allow them to remain long satisfied with a position which, to say the least, is always uncertain, if not in a majority of cases unsuccessful; and, if in the end they find that their best efforts to protect this sensitive organ from harm, have only been so many obstacles thrown in the way of the final success of the operation, it will not be the first discovery of the kind made in either branch of the healing art.

At all events it is to be hoped that the spirit of inquiry exhibited at the last meeting of the society, will not be allowed to subside, until some signal improvement have been made in this operation.

### ARTICLE VI.

*Morbid Sympathies of the Teeth.* By JOHN A. JOHNS, M. D., D. D. S. *Pleasant Grove, Va.*

